# Endoscopic ultrasound-guided jejunojejunostomy assisted by small bowel endoscopy for malignant bowel obstruction following digestive tract reconstruction

**DOI:** 10.1055/a-2977-7553

**Published:** 2026-10-28

**Authors:** Qilong Song, Jing Yang, Qian Xu, Xiaohua Hou, Heng Zhang, Yan Fan

**Affiliations:** 1 Department of Gastroenterology 577528 The Central Hospital of WuhanWuhanChina; 2 Key Laboratory for Molecular Diagnosis of Hubei Province 577528 The Central Hospital of WuhanWuhanChina; 3 Gastrointestinal Endoscopy and Clinical Research Center of Wuhan 577528 The Central Hospital of WuhanWuhanChina; 4 Department of Gastroenterology 4598 Union HospitalWuhanChina; 5 Department of Gastroenterology 577528 Huazhong University of Science and Technology Tongji Medical College Affiliated Wuhan Central HospitalWuhanHubeiChina

A 71-year-old man was hospitalized due to progressive dysphagia associated with nausea and vomiting for 2 months. His medical history included laparoscopic subtotal gastrectomy with gastrojejunostomy for gastric cancer performed 11 months prior to presentation. Pathological examination of the postoperative gross specimen revealed moderately to poorly differentiated adenocarcinoma of the stomach. A metallic stent was implanted across the anastomotic stricture caused by tumor recurrence.

Abdominal computed tomography (CT) scan showed localized small bowel dilatation and fluid accumulation ( Fig. 1 ), suggesting small bowel obstruction. However, despite aggressive medical management and placement of an intestinal obstruction catheter, the intestinal obstruction failed to resolve. Following a multidisciplinary team (MDT) discussion, endoscopic ultrasound (EUS)-guided jejunojejunostomy (EUS-JJ) assisted by single-balloon enteroscopy was performed under general anesthesia ( Video 1 , Fig. 2 a–f ). Postoperatively, the patient achieved unobstructed oral intake postoperative 48 hours. Four days after the EUS-JJ operation, abdominal CT scan showed satisfactory positioning of a lumen-apposing metal stent (LAMS) with no evidence of intestinal dilatation or fluid accumulation ( Fig. 3 ). The patient was followed up for 3 months with regular diet.

**Fig. 1:**
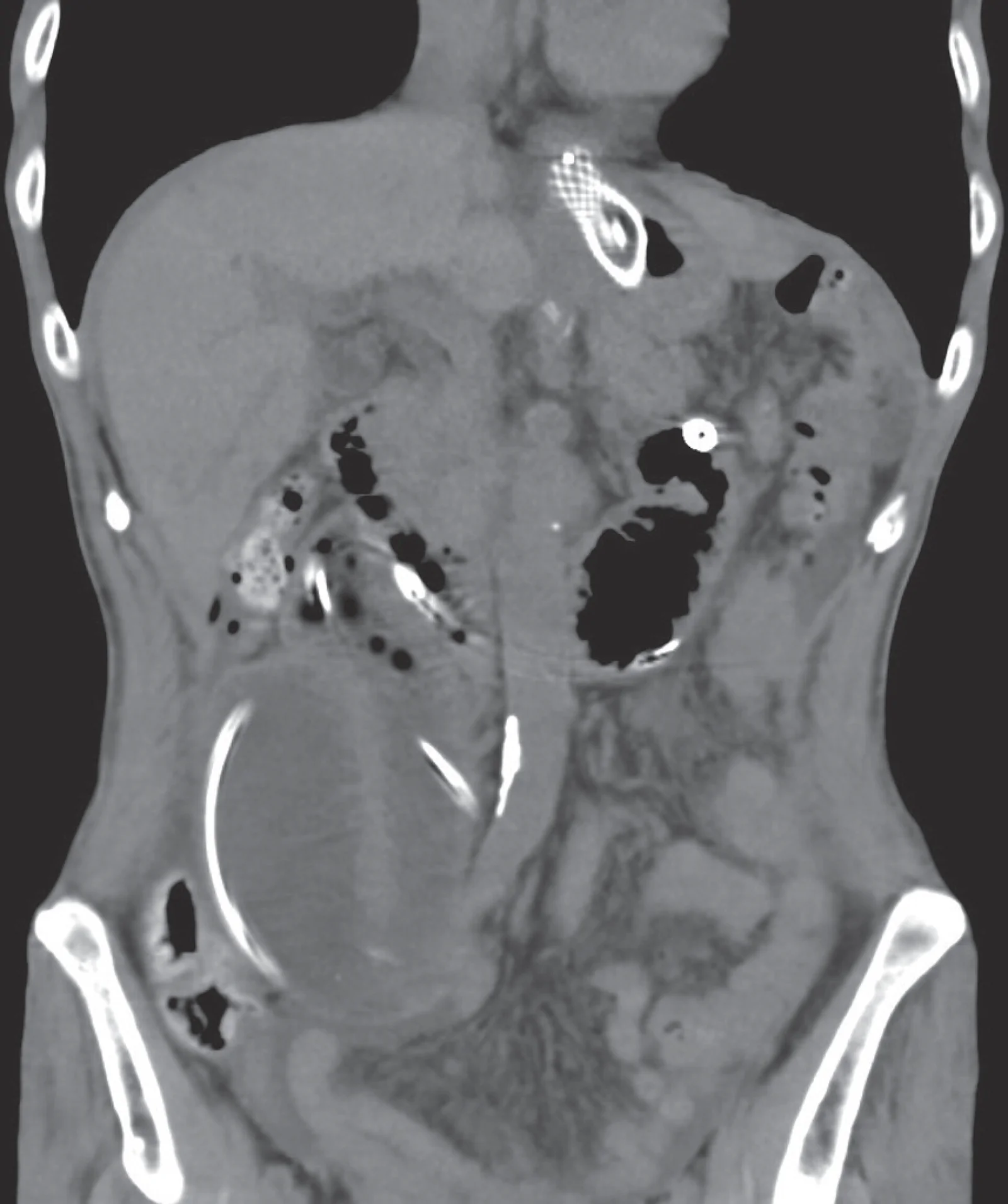
Preoperative coronal CT: dilated intestinal loop (yellow arrow).

**Video 1** EUS-guided jejunojejunostomy: Endoscopic and Fluoroscopic views.


**Fig. 2:**
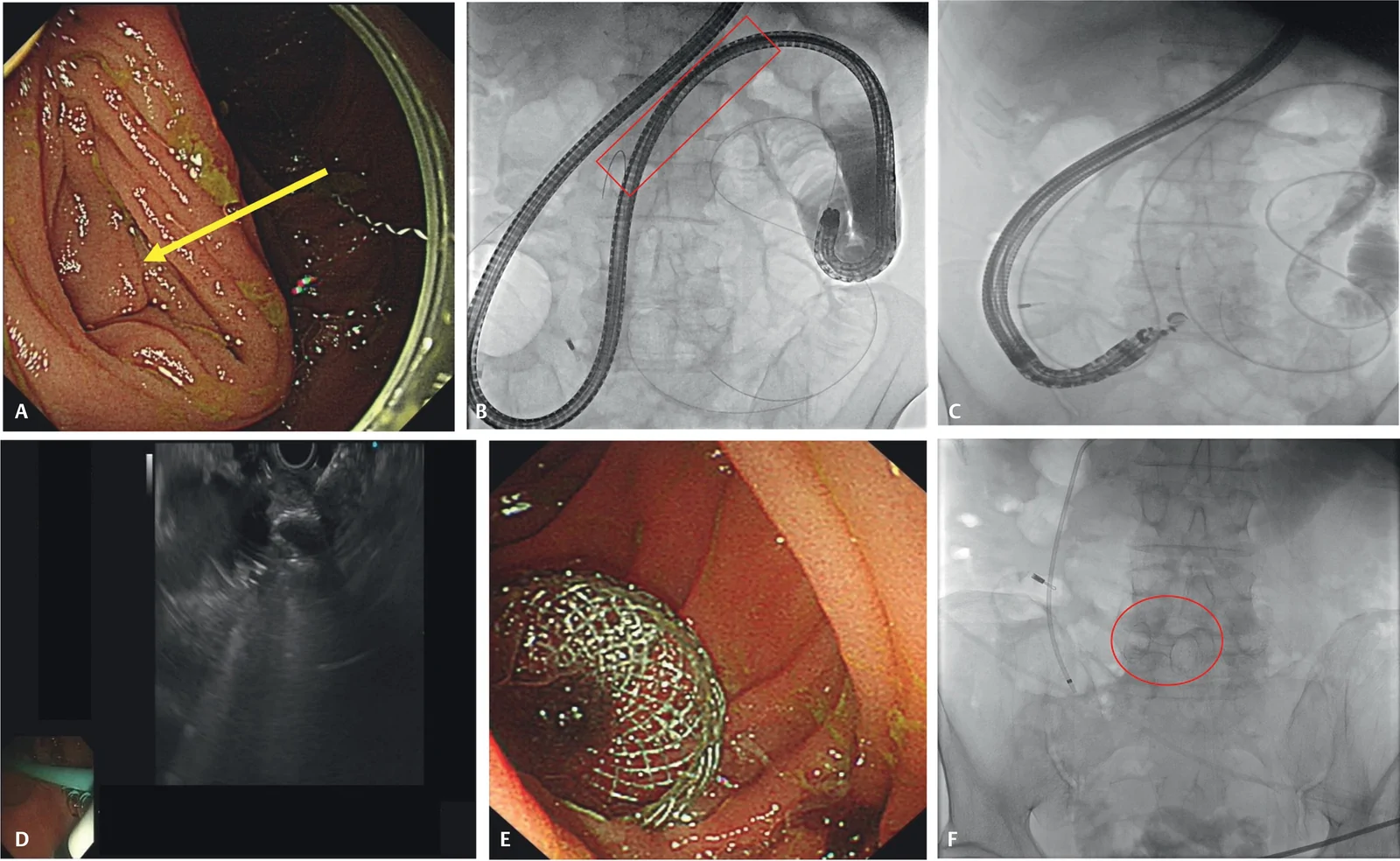
Procedure. **a** The enteroscope was advanced to the orifice of the efferent limb anastomosis (yellow arrow). **b** Enteroscopy confirmed multiple bowel luminal strictures (red box areas). The enteroscope was advanced across the strictured segment, and a guidewire was positioned in the distal bowel. **c** The endoscopic ultrasound was advanced to the planned anastomotic region. **d** The LAMS was deployed under endoscopic ultrasound guidance. **e** Endoscopic image of LAMS). **f** Radiographic imaging demonstrated that the LAMS stent bridged the afferent and efferent loops (red circle).

**Fig. 3:**
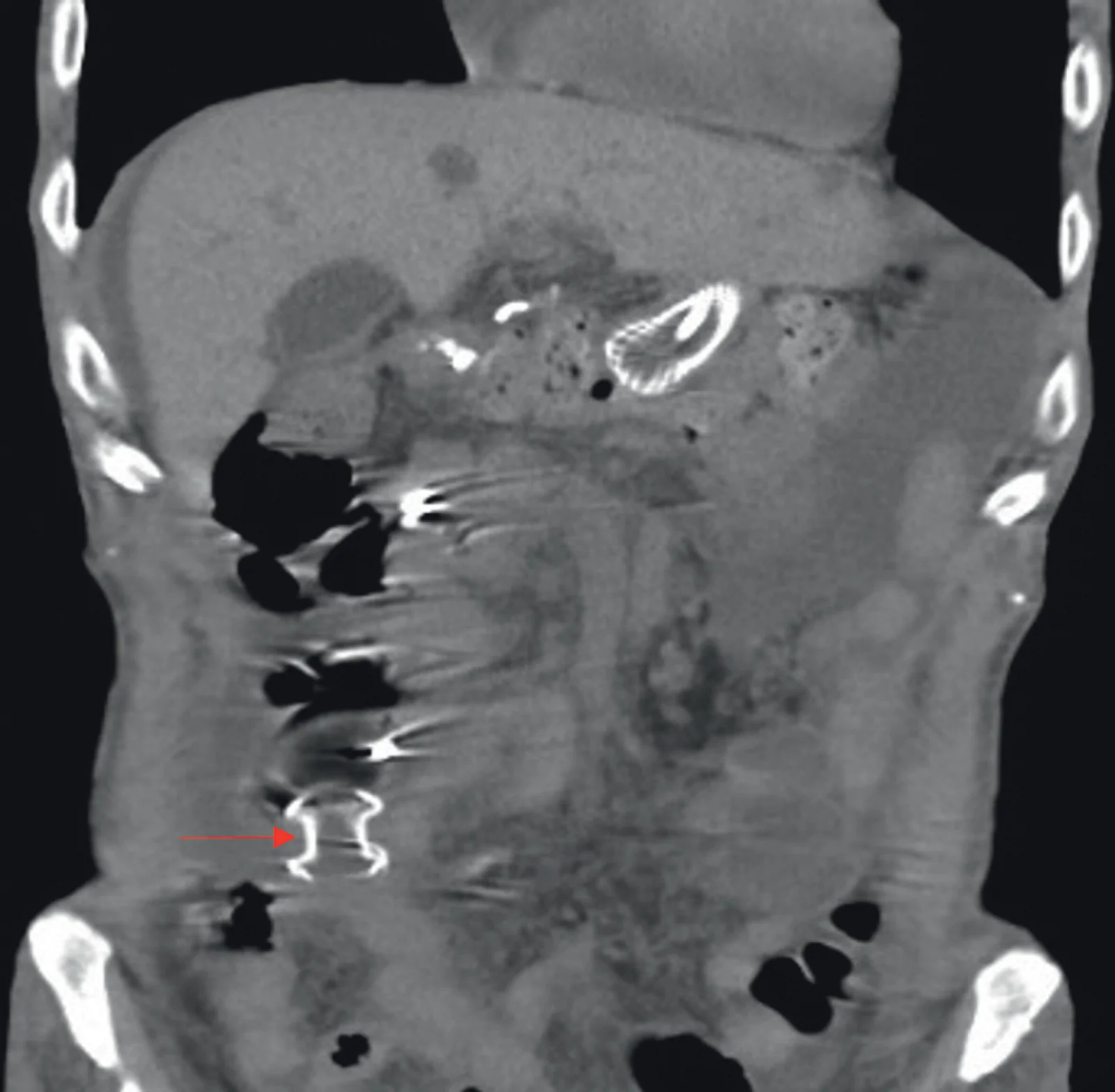
Postoperative coronal CT revealed that the LAMS (red arrow) was well positioned, with no dilated intestinal loops or intraluminal fluid collection.

EUS-JJ represents a therapeutic strategy for the alleviation of malignant bowel obstruction (MBO) in high-risk patients presenting with advanced cancer and significant comorbid conditions 1​ 2​ ​3​ . The anastomotic site for EUS-EE should be distant from the region of tumor recurrence, to prevent short-term recurrent obstruction. The integration of small bowel endoscopy and EUS not only helps identify the location of intestinal obstruction but also significantly enhances the success rate of jejunojejunostomy. This minimally invasive approach effectively resolves complex gastrointestinal obstruction caused by advanced tumors, substantially improves patients’ life quality with advanced malignancies, provides a window of opportunity for subsequent cytoreductive surgery and further oncological interventions, and ultimately prolongs overall survival 4​ .

Endoscopy_UCTN_Code_TTT_1AP_2AD

## Declaration of GenAI Use

The authors confirm that [AI tool- DeepSeek] was used solely for English language editing of this manuscript. No generative AI was used for data analysis, interpretation, or content generation. The authors confirm that no Generative AI was used in the preparation of this manuscript.
